# Utilizing quantitative immunohistochemistry for relationship analysis of tumor microenvironment of head and neck cancer patients

**DOI:** 10.1186/2051-1426-2-S3-P258

**Published:** 2014-11-06

**Authors:** Zipei Feng, Tarsem Moudgil, Allen Cheng, Christopher Paustian, Rieneke van de Ven, Christopher Dubay, Hong-Ming Hu, Tuan Bui, Tyler Hulett, Traci Hilton, Carlo Bifulco, Richard B Bell, Bernard A Fox

**Affiliations:** 1 Earle A. Chiles Research Institute, Providence Cancer Center; Department of Cancer Biology, Oregon Health & Science University, Portland, OR, USA; 2 Oral, Head and Neck Cancer Program and Clinic, Providence Cancer Center, OR, USA; 3 UbiVac, USA; 4 Molecular Microbiology and Immunology, OHSU, Portland, OR, Portland, OR, USA

## Background

Analysis of tumor-infiltrating immune cells using quantitative immunohistochemistry (IHC) has proved to be a powerful prognostic biomarker in colon cancer [1,2]. Similar observations have been made in patients with oral, head and neck squamous cell carcinoma (OHNSCC), where CD8 infiltration is associated with prolonged survival [3]. Recently, advancements are made in multiplex imaging and relationship analysis to better delineate suppressive mechanisms within the tumor microenvironment, which may direct immune interventions that augment tumor-specific immune response.

## Purpose

The purpose of this investigation was to apply multiplex immunohistochemistry and objective assessment techniques to identify biomarkers that correlate with HPV status, T cell infiltrate, and patient survival. Relationships analysis between immune markers and tumor cells will also be performed to examine the dynamic interactions that occur within the tumor microenvironment.

## Methods

92 subjects with biopsy-proven OHNSCC from different sub-sites underwent surgery with curative intent and were enrolled into this *prospective*, IRB approved protocol. Formalin-fixed-paraffin-embedded (FFPE) samples of patients' primary tumor or metastatic lymph nodes are obtained and stained for markers including CD4, CD8, CD137, CD163, interferon-gamma, arginase I, PD-L1, and class I, using the PerkinElmer Opal system. Images are scanned and analyzed using PerkinElmer Vectra system. Single stains are being done simultaneously using Ventana Benchmark XT and analyzed using Definiens platform.

## Results

**Figure 1:**
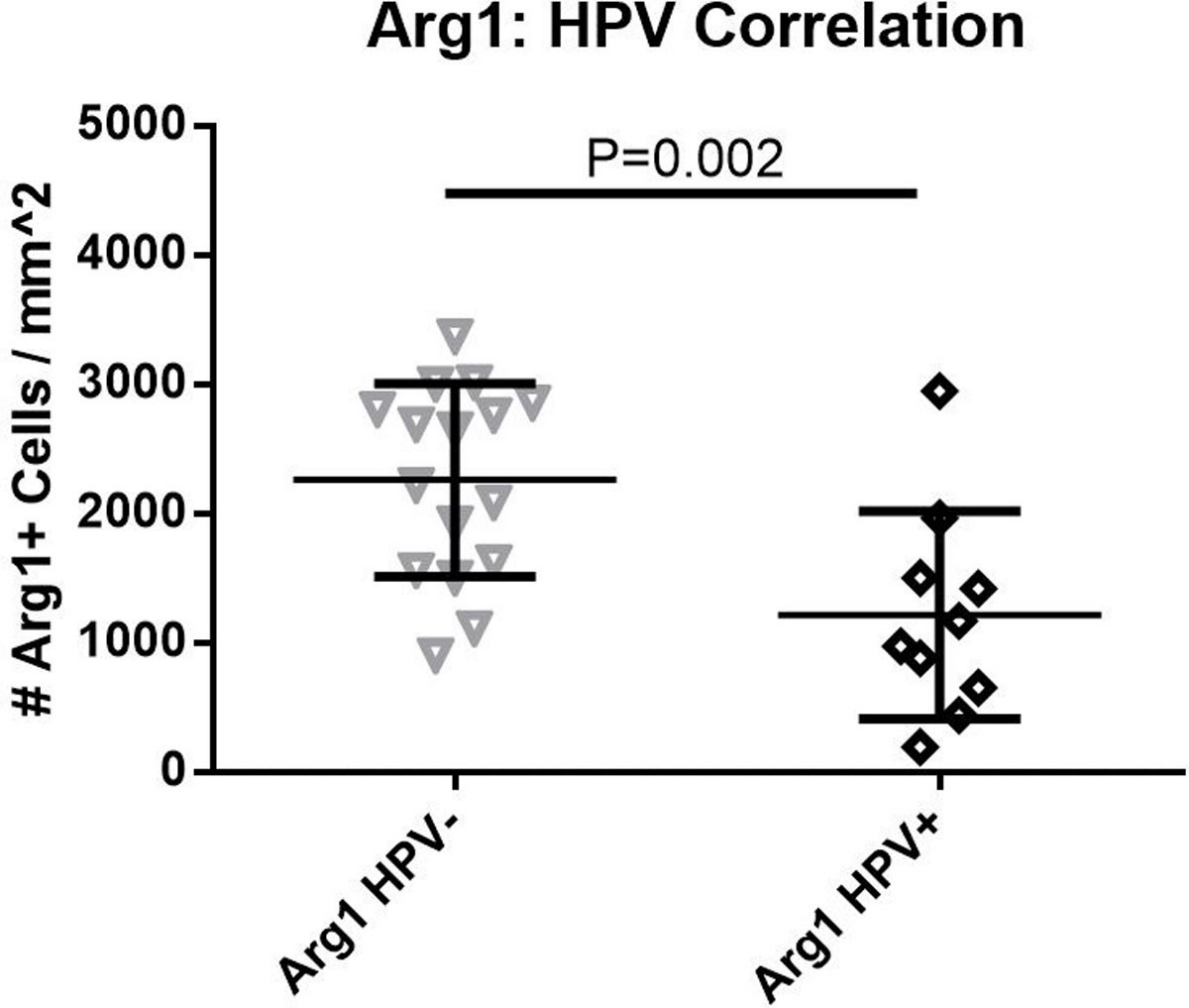
**Arg1: HPV correlation**.

**Figure 2:**
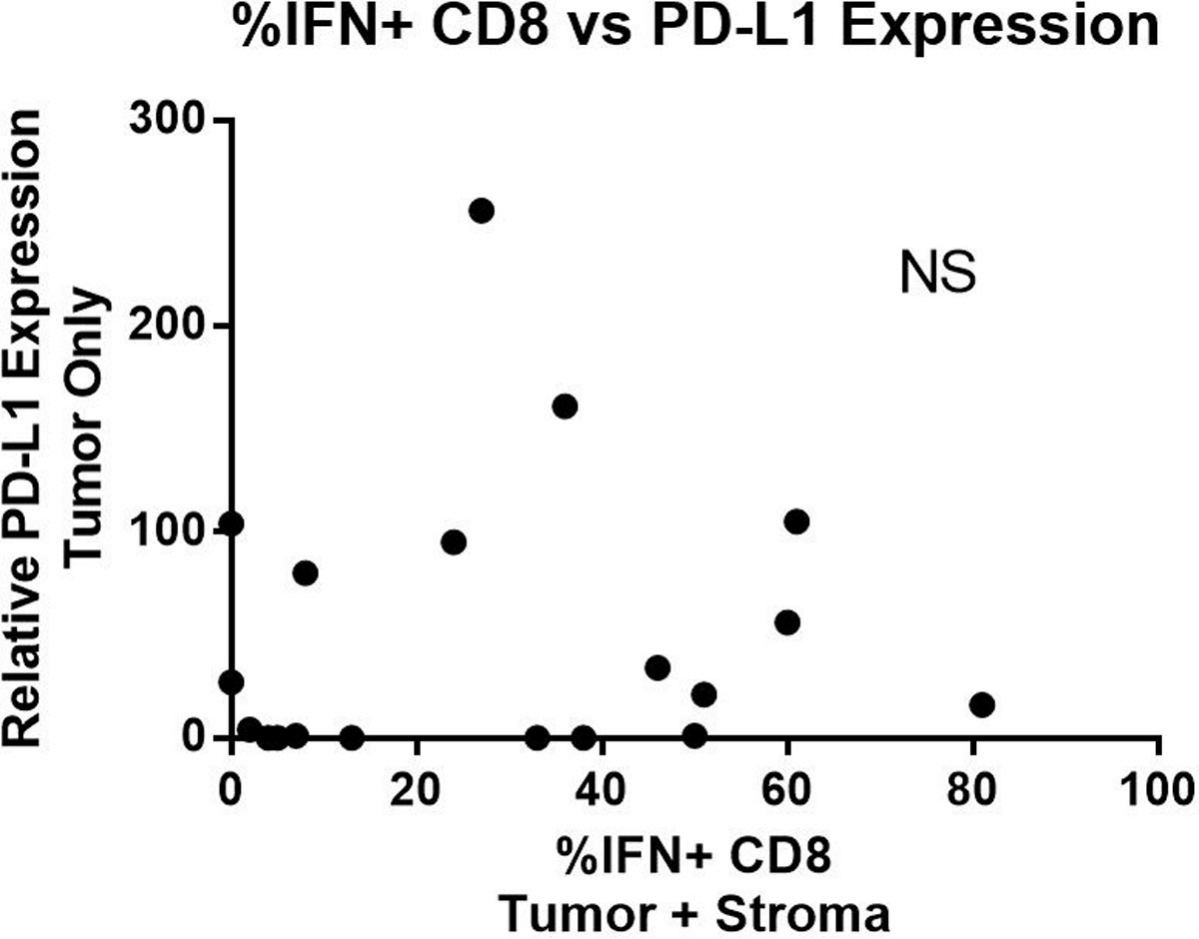
**% IFN+ CD8 vs PD-L1 expression**.

Preliminary results analyzed from 24 patients showed positive correlation between CD8 immune infiltrate within the tumor and HPV status (P = 0.05). Level of Arg1 within the tumor microenvironment showed a stronger correlation with HPV status (Figure 1), and inversely correlated with CD8 infiltrate (P = 0.03). Interestingly, the number of IFN-γ positive CD8 cells has no correlation with PD-L1 status in the subset of the patients that we have analyzed (Figure 2) - implying potential constitutive expression of PD-L1 in a subset of these patients.

## Conclusion

While still early, the technique is reproducible and can provide useful information on the relationships between various cells within the tumor microenvironment. Planned studies will assess the interplay between these markers in larger cohorts of patients with long-term follow-up, which aims to provide insights that may be exploited to develop novel therapeutic strategies that will improve outcomes of patients with OHNSCC.

## Support

Steve and Cindy Harder, Nancy and Wes Lematta, Robert W. and Elsie Franz, Lynn and Jack Loacker and The Chiles foundation.
